# Placental Abruption and Perinatal Mortality: Abnormal Placentation and Spontaneous Abortion as Contributors to Left Truncation Bias

**DOI:** 10.1111/ppe.70010

**Published:** 2025-06-05

**Authors:** Alan C. Kinlaw, Hillary L. Graham, Cande V. Ananth

**Affiliations:** ^1^ Division of Pharmaceutical Outcomes and Policy University of North Carolina School of Pharmacy Chapel Hill North Carolina USA; ^2^ Cecil G. Sheps Center for Health Services Research University of North Carolina Chapel Hill North Carolina USA; ^3^ Clinical Epidemiology Division, Faculty of Medicine at Solna Karolinska Institutet Stockholm Sweden; ^4^ Division of Epidemiology and Biostatistics, Department of Obstetrics, Gynecology, and Reproductive Sciences Rutgers Robert Wood Johnson Medical School New Brunswick New Jersey USA; ^5^ Department of Biostatistics and Epidemiology Rutgers School of Public Health Piscataway New Jersey USA; ^6^ Cardiovascular Institute of New Jersey Rutgers Robert Wood Johnson Medical School New Brunswick New Jersey USA; ^7^ Environmental and Occupational Health Sciences Institute (EOHSI) Rutgers Robert Wood Johnson Medical School New Brunswick New Jersey USA

**Keywords:** abnormal placentation, depletion of susceptible, left truncation, Monte‐Carlo simulation, perinatal mortality, placental abruption, selection bias, spontaneous abortion

## Abstract

**Background:**

Generally, studies in perinatal epidemiology restrict cohort entry to 20 weeks of gestation, but exposures and outcomes may occur earlier. This restriction may introduce left truncation bias.

**Objectives:**

To examine the impact of left truncation bias when estimating the causal effect of abruption on perinatal mortality in the context of abnormal placentation, with spontaneous abortion (SAB) as a censoring event.

**Methods:**

Through 80 Monte Carlo simulation scenarios based on realistic clinical assumptions, we estimated risk differences (RD), risk ratios (RR) and bias parameters for the abruption–perinatal mortality association.

**Results:**

Censoring by SAB ranged from 5.6% to 7.6% across simulation setups. The risk of mortality was overestimated in observable (left‐truncated) data at ≥ 20 weeks compared to an unobservable cohort starting follow‐up at placental implantation (conception cohort). Underestimation of risks was stronger among abruption pregnancies. RDs for the abruption‐mortality association were biased by +1% to +3% among conceptions with normal implantation and by +5% to +43% among abnormal placentation. Due to the disproportionate underestimation of mortality among nonabruption pregnancies, RRs were overestimated by 1.1 to 1.2‐fold for normal implantations and by 1.1 to 8.4‐fold for abnormal implantations.

**Conclusions:**

The findings of this simulation study highlight the critical importance of placentation in successful pregnancy. Abnormal placentation has profound consequences for unsuccessful pregnancies, remarkably increasing the risks of early losses, placental abruption and other obstetrical complications. This study underscores that left truncation can bias the abruption–perinatal mortality association, differentially by whether the placentation was normal or abnormal. However, defining the causal question regarding the abruption–perinatal mortality association requires consideration of the target population, which may include all conceptions. In studies of these effects, outcome follow‐up capability may introduce left truncation bias. We do not prescribe one analytic approach to account for left truncation, but rather, the approach should be guided by the causal question.

## 1 Background

**FIGURE 1:**
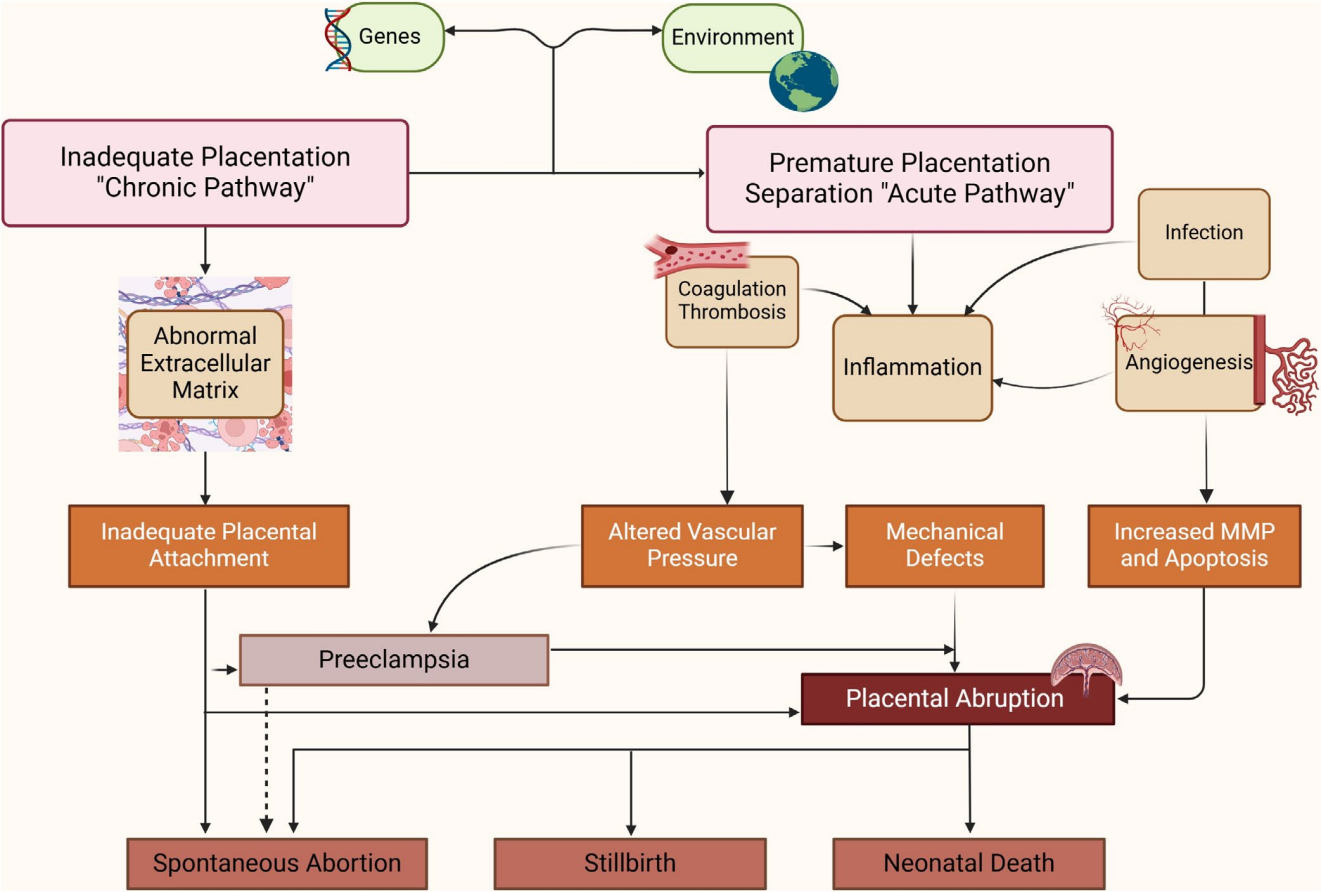
A conceptual framework describing acute and chronic pathways between placental health, placental abruption, and perinatal outcomes, including mortality. Purple indicates the two processes that initiate placental separation. Green indicates the pathophysiological mechanisms as the consequences of placental separation. Yellow indicates the cellular changes as downstream consequences of placental separation. Orange indicates the occurrence of preeclampsia due to extracellular matrix remodelling, coagulation, and thrombosis. Bright red indicates the occurrence of placental abruption (the chief exposure in this study). Dark red indicates the adverse perinatal consequences of placental abruption (the primary outcomes). We show the dotted line from preeclampsia to SAB to denote that we are not explicitly accounting for this pathway in this study.

Placental abruption, a devastating obstetrical complication, is diagnosed when placental detachment occurs prematurely and precedes the delivery of the foetus [1]. While the origins of abruption remain unknown, we have hypothesised that the condition is the end result of two pathophysiologic mechanisms: [2, 3] inadequate placentation, a process that occurs around the stages of placental implantation early in pregnancy [4], and premature placental detachment, a process that happens closer to labour and delivery (Figure 1) [3]. Accumulating evidence from clinical observations, epidemiologic data and histologic findings suggests that abruption from inadequate placentation and premature placental detachment pathways largely indicates a chronic process early in pregnancy (which occurs in 80%–85% of all abruptions) and an acute process that occurs closer to delivery, respectively [5, 6, 7, 8].

Placentation plays a crucial role in ensuring a successful pregnancy. Any abnormalities in the placental implantation process can have devastating consequences, resulting in pregnancy loss on one end to the manifestation of pathological complications with implications on health along the life course [9]. Placental abruption, one such pathology from abnormal placental implantation, confers disproportionately high rates of perinatal mortality [10, 11, 12, 13, 14, 15, 16], and evidence for this association comes exclusively from studies that are restricted to populations of stillbirths and live births, occurring at 18–20 weeks or later in gestation. Understandably, this is primarily because the exposure (abruption) is difficult, if not impossible, to ascertain until the latter half of pregnancy and at delivery. Although valid for estimating the association between clinically recognised abruption and perinatal mortality, these studies may mask the extent of overall harms conferred by abruption on the growing foetus, including unobservable events before 20 weeks of pregnancy.

### 1.1 Selection Bias Due to Left Truncation

Observational studies can provide persuasive evidence regarding causal exposure effects. However, bias due to selection can compromise the validity of associations and threaten their generalisability [17, 18]. One form of selection bias is bias due to left truncation or differential delayed entry into the cohort [19, 20, 21]. Another conceptualisation of left truncation arises in the setting of missing person‐time [22]. This bias occurs when people are exposed before entry into the cohort or when the outcome or a competing event of the outcome occurs before recruitment. Left truncation bias is common in studies in perinatal epidemiology since the gestational age at entry for inclusion into a cohort (time 0) is often set at 20 weeks gestation, but exposures and outcomes may occur earlier. The goal of our simulations is to demonstrate how different the effect of abruption would be on mortality had the association been tested in a ‘conception cohort’ (i.e., ‘unobservable’ data, no left truncation bias) versus a ‘birth cohort’ with delayed entry at 20 weeks in gestation (i.e., observable data, with potential left truncation [23]).

Of course, for a ‘birth cohort’ study with an implicit fixed‐left truncation [22] at 20 weeks, left truncation bias can occur if the investigators fail to restrict their interpretation of results to the target population of pregnancies that have survived past the window of spontaneous abortion (SAB) (i.e., at < 20 weeks). Since SAB is impacted by largely immeasurable early pregnancy factors such as abnormal placentation and abruption onset, left truncation *bias* can be introduced if the interpretation of results extends earlier into pregnancy. We recognise that a smaller proportion of abnormal placentation may result in complications that warrant an induced abortion. Since these abortions are likely independent of abruption onset, we do not focus on induced abortions in this study.

### 1.2 Motivation and Research Question

This study aims to quantify abruption's effect on survival from early gestation (in the context of SAB) through pregnancy duration to delivery (live or stillbirth), and neonatal outcomes within the first month of life—not just the survival of foetuses past 20 weeks gestation. We posit that the critical causal question is to estimate the total effect of abruption (from inadequate placentation or premature placental detachment, Figure 1) on stillbirth or neonatal mortality. The implicit assumption that underlies this motivation relates to the ‘depletion of susceptibles’ theory, in that inadequate placentation leads to differential rates of SAB in the presence and absence of unrecognised abruption. This differential left truncation may obscure our understanding of the mechanisms through which abruption impacts SAB and perinatal mortality. Clarifying these mechanisms and their corresponding magnitudes of effect could help us bound the potential impact of exposures that might interfere with placentation or of interventions to manage abruption through pregnancy, even before 20 weeks.

Subsequently, the foetuses‐at‐risk conceptualisation [24, 25] is especially helpful for studying the ongoing threats posed by abnormal placentation and abruption throughout pregnancy and into the neonatal period. This simulated cohort study follows conceptions from implantation through the neonatal period. Since a clinical abruption diagnosis is only observable at ≥ 20 weeks in gestation, we examine the impact of left truncation bias when estimating the placental abruption–perinatal mortality association.

## 2 Methods

We undertook a comprehensive Monte–Carlo simulation analysis to evaluate the impact of left truncation bias on the causal effect of abruption onset (*X*) on perinatal mortality (*Y*).

### 2.1 Simulation Study

**FIGURE 2:**
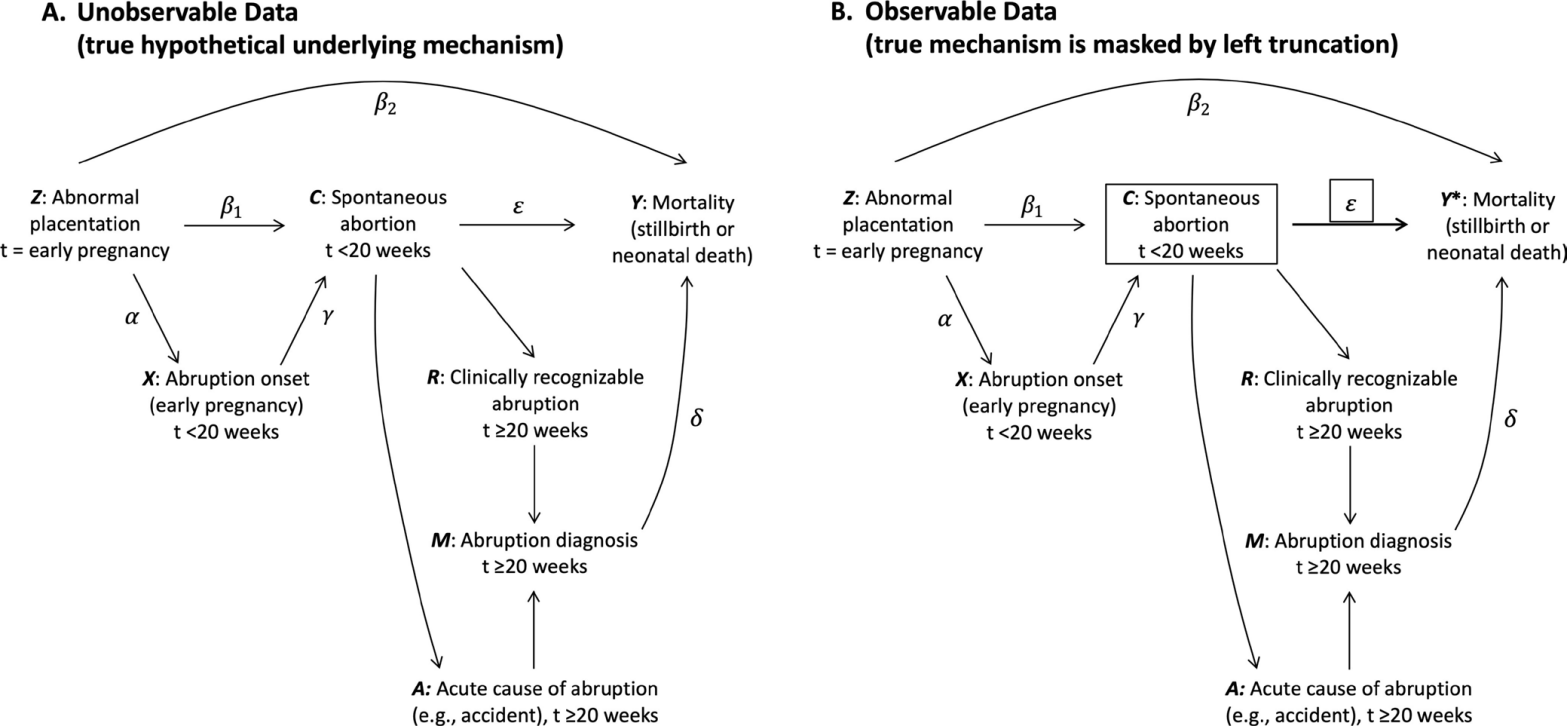
Causal diagram representing associations in the simulated data between abnormal placentation (*Z*), placental abruption onset (*X*), spontaneous abortion (*C*), placental abruption diagnosis (*M*) and mortality (*Y*). Panel (A) represents the unobservable data in which spontaneous abortions are counted; panel (B) represents classically observable data in which investigators implicitly condition on spontaneous abortion (indicated by the circle around *C*: Spontaneous abortion occurring at < 20 weeks) by restricting a study to pregnancies lasting at least 20 weeks gestation since mortality in the naïve data (Y*) could only arise from stillbirth (occurring 20–42 weeks) or neonatal death (occurring from birth to 28 days). Under the assumption of unmeasured confounding associated with spontaneous abortion and mortality (not shown), conditioning on spontaneous abortion can induce collider bias and alter the ability to estimate the total effect of an exposure on an outcome (rather than estimating decomposed effects, which require other nuanced assumptions).

We performed a Monte–Carlo simulation study using several published studies to base our data‐generating mechanism and estimated risks, risk differences (RD), risk ratios (RR) and bias parameters for the association between abruption and SAB across various simulation scenarios. Figure 2 shows the causal diagram underpinning our simulation setups; Table 1 enumerates the assumptions regarding the association between the study variables, including relevant citations. In the supplement, we provide a spreadsheet that details all inputs for every simulation scenario and a SAS macro that allows the reader to explore alternative ranges of assumptions than ours.

**TABLE 1:** Summary of simulation parameters and assumptions.

Parameter	Causal factors	Probability function(s)	Range of assumed input values across simulation setups	Additional notes	Relevant citations
*Z*: Abnormal placentation (Early pregnancy)	None	$\mathrm{Pr}\left(Z\right)=\alpha$	$\alpha =\mathrm{Pr}\left(X\right):\left(0.1,0.2^{\text{a}}\right)$		No published data
*X*: Abruption onset (*t* < 20 weeks)	*Z*	$\mathrm{Pr}\left(X\right)=\left\{\begin{array}{ll} U\left(0.005,0.050\right), & Z=1\\ U\left(0.001,0.020\right), & Z=0 \end{array}\right.$	Same across simulation setups	Average RR(*X*|*Z*) = 3.6 *Z* is the only causal factor for *X* Probabilistic association between *Z* and *X*	No published data
*C*: Spontaneous abortion (< 20 weeks)	*Z*, *X*	$\mathrm{Pr}\left(C\right)=\exp\left(\alpha_{0}+\alpha_{1}Z+\alpha_{2}X+\alpha_{3}\mathrm{XZ}\right)$	$\exp\left(\alpha_{0}\right)=\mathrm{Pr}\left(C|Z=0,X=0\right):\left(0.05\right)$ $\exp\left(\alpha_{1}\right)=\mathrm{RR}\left(C|Z\right):\left(2,4,6\right)$ $\exp\left(\alpha_{2}\right)=\mathrm{RR}\left(C|X\right):\left(2,4.5\right)$ exp(*α*_3_): (1) (no heterogeneity of RRs)		Wilcox et al. [26] Jones and Jerman [27]
*M*: Abruption diagnosis (≥ 20 weeks of pregnancy)	*X*, *C*	$M=\left\{\begin{array}{ll} \mathrm{mi}\text{ssin}g, & C=1\\ X, & C=0 \end{array}\right.$	Same across simulation setups.	Abruption diagnosis deterministically associated with joint distribution of abruption onset and spontaneous abortion	Saftlas et al. [28] Sheiner et al. [29] Tikkanen [30] Ananth et al. [31] Downes et al. [32]
*Y*: Mortality (Stillbirth or neonatal death)	*Z*, *C*, *M*	$\mathrm{Pr}\left(Y\right)=\left\{\begin{array}{ll} 0, & C=1\\ \exp\left(\alpha_{0}+\alpha_{1}M+\alpha_{2}Z+\alpha_{3}\mathrm{MZ}\right), & C=0 \end{array}\right.$	$\exp\left(\alpha_{0}\right)=\mathrm{Pr}\left(Y|M=0\;Z=0,C=0\right):\left(0.0082,0.0120^{\text{a}}\right)$ $\exp\left(\alpha_{1}\right)=\mathrm{RR}\left(Y|M,C=0\right):\left(14.5,9.9^{\text{a}}\right)$ $\exp\left(\alpha_{2}\right)=\mathrm{RR}\left(Y|Z,C=0\right):\left(2,4\right)$ exp(*α*_3_): (1) (no heterogeneity of RRs)	Death partially deterministically associated with spontaneous abortion; among nonspontaneous abortions, death depends on abnormal placentation and abruption diagnosis	Ananth and Wilcox [4] Ananth and VanderWeele [33]
*S* ^a^: In utero mortality (Stillbirth)	*Z*, *C*, *M*	$\mathrm{Pr}\left(S\right)=\left\{\begin{array}{ll} 0, & C=1\\ \exp\left(\alpha_{0}+\alpha_{1}M+\alpha_{2}Z+\alpha_{3}\mathrm{MZ}\right), & C=0 \end{array}\right.$	$\exp\left(\alpha_{0}\right)=\mathrm{Pr}\left(Y|M=0,Z=0,C=0\right):\left(0.0053^{\text{a}},0.0077^{\text{a}}\right)$ $\exp\left(\alpha_{1}\right)=\mathrm{RR}\left(Y|M,C=0\right):\left(14.5^{\text{a}},9.9^{\text{a}}\right)$ $\exp\left(\alpha_{2}\right)=\mathrm{RR}\left(Y|Z,C=0\right):\left(2^{\text{a}},4^{\text{a}}\right)$ exp(*α*_3_): (1^a^) (no heterogeneity of RRs)	Stillbirth partially deterministically associated with spontaneous abortion; among pregnancies that survive past 20 weeks, stillbirth depends on abnormal placentation and abruption diagnosis	Ananth and Wilcox [4] Ananth and VanderWeele [33]

Abbreviations: exp, anti‐logarithm with base *e*; Pr, probability; RR, risk ratio.

^a^ Value used only in sensitivity analyses.

We focus on two primary factors that impact the survival of implanted conceptuses through the neonatal period—inadequate placentation and abruption. Inadequate placentation (unobservable) is a precursor to abruption [34, 35, 36, 37], and both factors develop early in pregnancy and cause an increased risk of SAB, stillbirth (at ≥ 20 weeks in pregnancy), and neonatal death (within 28 days after live birth). Abnormal placentation and abruption can originate as early as implantation of the conceptus (we theorise that abnormal placentation precedes an abruption), although early abruption is not yet recognisable. A clinical diagnosis of abruption is not made until later in pregnancy—often at delivery but sometimes detected on prenatal ultrasound [6, 38]. In the simulations, we encoded abruption at two distinct time points—onset (early during pregnancy) and diagnosis (typically in the latter half of pregnancy and delivery).

We adopted the foetuses‐at‐risk conceptualisation [25, 39] to study the risks associated with abnormal placentation and abruption throughout pregnancy and its effect on the neonatal period. As a result, in this simulated cohort study, conceptuses are followed from implantation until the end of the neonatal period, at which point their outcome (*Y*) is classified as perinatal mortality (stillbirth or neonatal death) or having survived the neonatal period.

Notably, in Figure 2, *β*_2_ represents the association between abnormal placentation (*Z*, originating early in pregnancy) and risk of outcome (*Y*) components representing perinatal mortality. This association is separate in our simulation setup to suggest that there are plausible causal mechanisms through which abnormal placentation could increase the risk of perinatal mortality outside the path mediated by abruption. It also implies that any (unmeasured) mediating factors along edge *β*_2_ are independent of censoring by SAB, which is a simplifying assumption since many unmeasured or unknown factors associated with placentation and mortality may be associated with SAB.

Turning towards the estimation of the association of interest, we acknowledge a final assumption: that the association of primary interest (i.e., the total effect of abruption onset *X* on mortality *Y*) involves two causal paths in the unobservable data, *X*➔*C*➔*Y* and *X*➔*C*➔*M*➔*Y*, which are both blocked in left‐truncated observable data. Edge *ε* affirms the assumption encoded in the simulation setup that SAB precludes the occurrence of stillbirth or neonatal death. Based on the DAG (Figure 2), it is necessary to condition on abnormal placentation (*Z*) to estimate $\hat{\alpha_{1}}$ and $\hat{\alpha_{3}}$ from the log‐binomial model $\log\left[\hat{\mathrm{Pr}}\left(Y|X,Z\right)\right]=\hat{\alpha_{0}}+\hat{\alpha_{1}}X+\hat{\alpha_{2}}Z+\hat{\alpha_{3}}\mathrm{XZ}$. We, therefore, present results stratified by abnormal placentation (*Z*) to account for potential confounding and scale‐dependent modification by abnormal placentation on the association between abruption onset (*X*) and mortality (*Y*). We generated data for a primary analysis of 10 simulation setups (Table 1 and Supplementary Figure S1 for details), and sensitivity analysis using 30 additional simulation setups with other combinations of assumptions (Table 1, Figure S2).

Finally, for multiple parameter estimates, we quantified the following biases:

- The absolute bias of risk of death in each stratum of *Z*, *X* (risk_observed_—risk_unobserved_).
- The absolute bias of RD for *Y*|*X* in each stratum of *Z* (RD_observed_—RD_unobserved_).
- The relative bias of RR for *Y*|*X* in each stratum of *Z* (RR_observed_/RR_unobserved_).

### 2.2 Sensitivity Analysis: In Utero Mortality as an Outcome

To explicitly consider one component of perinatal mortality, we conducted a sensitivity analysis of 40 additional simulation setups to consider in utero death (stillbirth only, denoted as *S*) separately from perinatal mortality in the primary analysis (Table 1, Figures S3 and S4). We simulated 200 cohorts in each setup, each with 100,000 implanted conceptuses.

## 3 Results

### 3.1 Simulation Study

**FIGURE 3:**
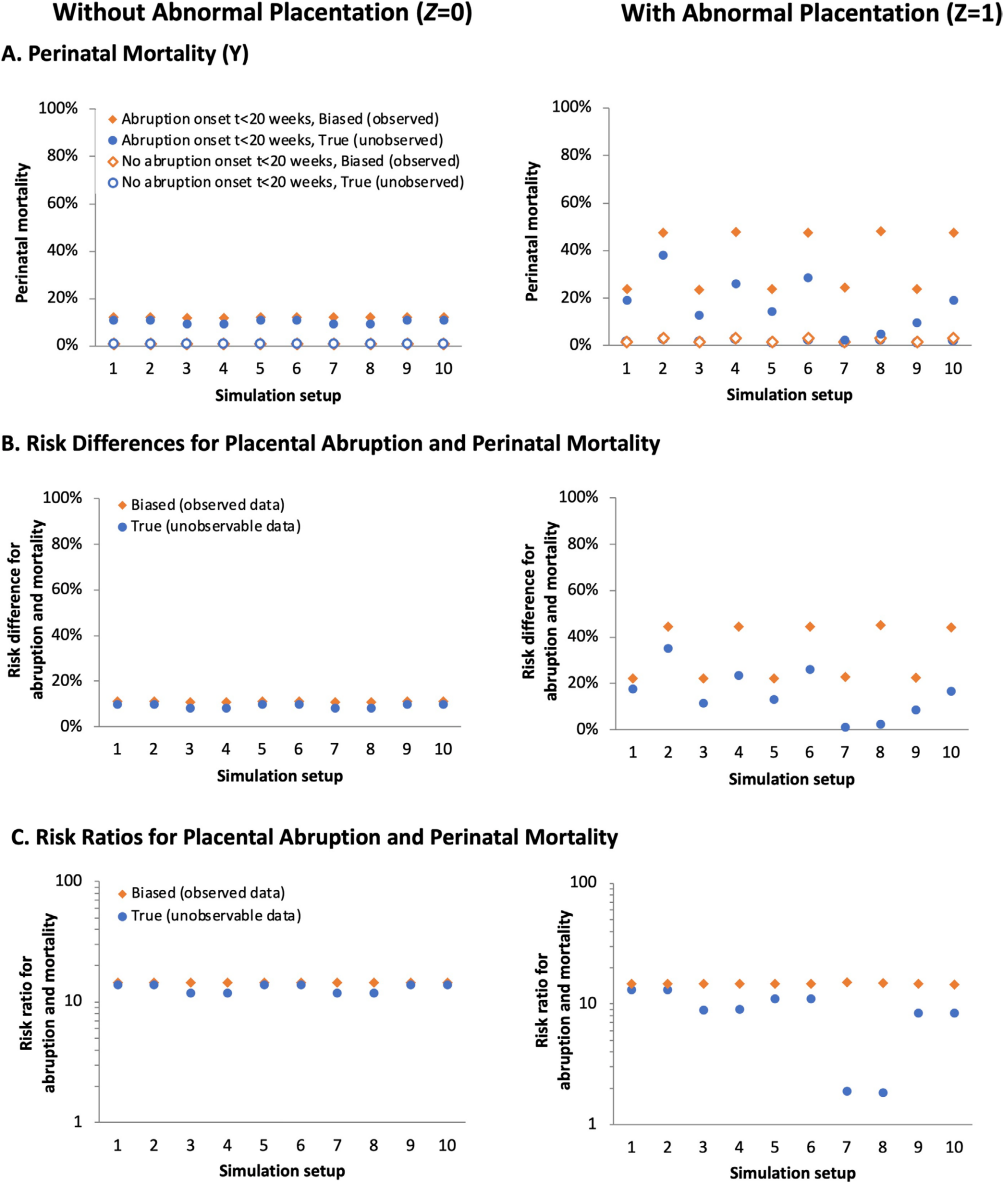
Risks of perinatal mortality (panel A), risk differences (panel B), and risk ratios (panel C) across simulation setups in the primary analysis, stratified by abnormal placentation status. Perinatal mortality includes stillbirth (at ≥ 20 weeks) or neonatal death (within the first 28 days); parameter combinations across simulation setups 1 to 10 are provided in Figure S1.

Across simulation setups, censoring due to SAB (C) ranged from 5.6% to 7.6%. In the analysis of the observed (left‐truncated) data compared to unobservable data (the entire cohort that would have been observed had follow‐up begun at placental implantation), the risk of perinatal mortality was overestimated for pregnancies both with and without abruption (Figure 3). In the setting of the normally implanted placenta (*Z = 0*, left panels), the estimated mortality risk was higher for pregnancies with abruption (*X = 1*, solid circles) than for those without abruption (*X = 0*, open circles). In the presence of abnormal placentation (*Z = 1*, right panels), the mortality risk was much higher for pregnancies with abruption onset and had a wider distribution in the unobserved data compared to the observed data.

Fewer deaths were observed in the biased data, and unobserved deaths were differentially more common among pregnancies with abruption onset. RRs were consistently overestimated in the biased data, driven by the exceedingly low referent risk of mortality in the observed data (i.e., only among pregnancies that did not result in SAB). The bias of RRs was prominent across all scenarios. However, the bias in RDs (on the absolute scale) was lowest in two types of settings: (i) for normally implanted placentas when the total effect of abruption onset (*X*) on SAB (*C*) was weakest (RR = 2); and (ii) for abnormally implanted placentas when that RR(*SAB*|*X*) was weakest (RR = 2) and the total effect of abnormal placentation (*Z*) on mortality (*Y*) was strongest (RR = 4).

**FIGURE 4:**
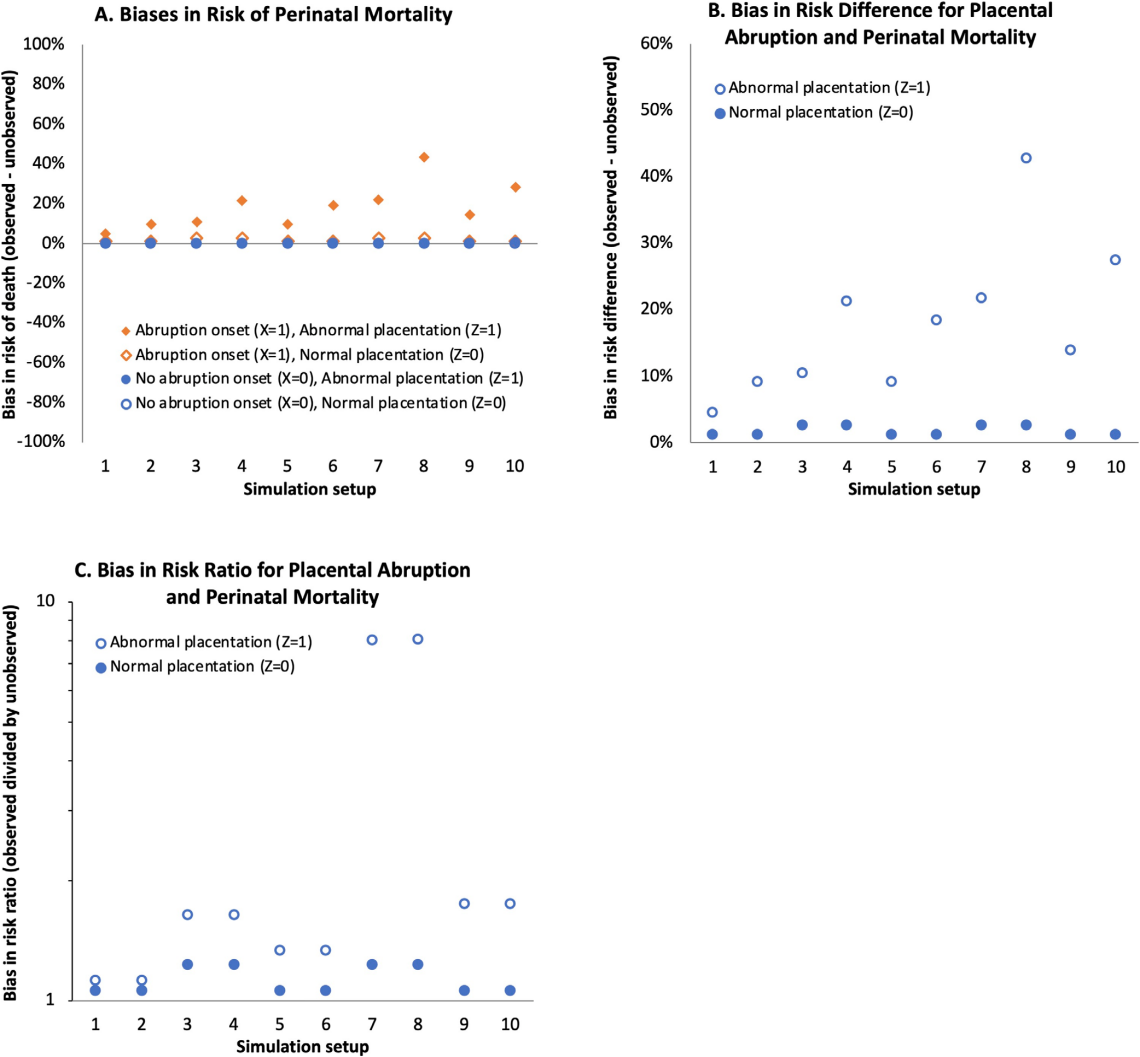
Bias estimates: Bias in risks of perinatal mortality (panel A), risk differences (panel B), and risk ratios (panel C) across simulation setups, stratified by abnormal placentation status.

Biased estimates for the risk of perinatal mortality depended on abruption onset and further varied depending on the prevalence of abnormal placentation (Figure 4A). The absolute bias in RDs was overestimated by 1% to 3% among normally implanted placentas (Figure 4B). Among abnormally implanted placentas, the absolute bias of the RD was more extreme, with overestimation ranging from 5% to 43%. RRs were also overestimated—by 1.1 to 1.2‐fold for normal implantations and by 1.1 to 8.0‐fold for abnormal implantations (Figure 4C). Although the measures of absolute risks and RDs (and their bias measures) varied across scenarios (from near‐zero to extreme bias), RRs and their bias appeared more similar across simulation setups.

### 3.2 Sensitivity Analysis

Results for the 30 sensitivity analysis simulation setups 11–40 yielded similar overestimation patterns to the primary analysis (Figures S5 and S6). Additionally, for the sensitivity analyses restricted to in utero death (setups 41 to 80), biases followed a similar pattern to those observed for the composite outcome: among normal implantation, RDs were biased by 1% to 2%, and RRs were overestimated by 1.1 to 1.2 among normal implantation; among abnormal implantation, RDs were biased by 3% to 28% and RRs were overestimated by 1.1 to 8.1 (Figures S7 and S8).

## 4 Comment

### 4.1 Principal Findings

Through extensive simulations and empirical analysis, we show that restricting the study to a ‘birth cohort’ rather than a ‘conception cohort’ biases effect measures (both on additive and multiplicative scales) of the association between abruption and mortality when the target population is all conceptions. The mortality risk is overestimated in the observed (left‐truncated) data starting at ≥ 20 weeks compared to the unobservable conception cohort starting at the stages of placental implantation. Specifically, the bias in RDs yielded overestimates as strong as +3% among normally implanted placentas and +43% among abnormally implanted placentas; RRs, on the other hand, were overestimated by between 1.1 and 8.0 times the true RR.

#### 4.1.1 Strengths of the Study

The study's strengths include realistic simulation scenarios, validated simulation parameter assumptions from prior studies, and comprehensive sensitivity analyses.

#### 4.1.2 Limitations of the Data

A limitation of this study is missing or immeasurable data on exposures, outcomes and key health indicators during pregnancy, especially in early pregnancy. In the simulation portion of our research, we generated data based on prior literature to examine the association between abruption and mortality. Our data generation necessarily carries simplifications we assumed to carry out the analysis. For example, we assessed abnormal placentation and abruption as dichotomous variables and restricted the timescale at which they could arise, simplifying some of these conditions' complexities [7]. We also posited several deterministic (or at least partially deterministic) associations instead of data that could add nuance to those assumptions. Finally, as shown in the DAG (Figure 2), we assumed that (i) all births with abruption had onset (*X*) during early pregnancy (implantation stage); and (ii) there is no direct effect of abruption onset (*X*) on abruption diagnosis (*M*) without mediating action by SAB (*C*). There may be another path directly from *X* to *M* (not mediated by *C*) in which the onset of conditions that may lead to abruption is associated with an increased risk for clinically recognised abruption later in pregnancy and independent of the occurrence of SAB. Our simulations do not address this. This could give rise to unmeasured confounding bias in ‘traditional’ studies of *M*➔*Y* (among pregnancies surviving at ≥ 20 weeks in gestation) due to abruption onset differences that may be independent of SAB [40].

#### 4.1.3 Interpretation

Studies have documented disproportionately increased rates of perinatal mortality among abruption births [4, 11, 16, 30, 41, 42]. We designed this study to address the intersection among abruption, its primary cause—abnormal placentation—and early pregnancy loss, and how their occurrence may provide new insights into our understanding of mortality among foetuses and neonates conferred by abruption. Studies of abruption and perinatal mortality are typically restricted to populations of stillbirths and live births occurring after 20 weeks of pregnancy. Understandably, this is primarily because abruption is challenging to ascertain until later in pregnancy and closer to, or at the time of, delivery—especially in real‐world settings. Although valid for inference of the effect of abruption on perinatal mortality, these studies may mask an understanding of the overall harms conferred by abruption on the growing foetus, including before 20 weeks of pregnancy.

There is a difference between the *X*➔*Y* association estimated from the unobserved simulated data (‘What could the underlying effect of *X* on *Y* be?’) and the *M*➔*Y* association encoded in the simulated data (‘among pregnancies surviving 20 weeks, what was the association between abruption and perinatal mortality, *Y*?’). These associations reflect different target populations and thus cannot be directly compared as bias indicators. When comparing these estimates, the relevant question is, ‘How did potential misspecification of the target population obscure our understanding of how harmful abruption in early pregnancy can be for implanted conceptuses?’ On the other hand, the difference between estimates of the *X*➔*Y* association in the unobserved simulated data and the *X*➔*Y* association in the observed data (after the competing events are removed) does represent a bias due to left truncation. ‘Rather than focusing only on the impact of abruption on survival starting at 20 weeks, what if we instead account for SAB when estimating the mortality associated with abruption from its time of onset?’ Abruption is often unpredictable—particularly when triggered by an acute insult to the placenta—resulting in the ‘premature placental separation’ pathway (Figure 1). However, a nontrivial proportion of such abruptions have their origins early in pregnancy (before 20 weeks in gestation), are subclinical, and (i) result in SAB s or (ii) manifest their symptoms later in gestation. In the former, the pregnancy is terminated with an aborted foetus; in the latter, the only intervention is an imminent delivery. Since we are examining the role of abnormal placentation in this study of abruption and mortality, we believe studying events before 20 weeks is critical to frame an appropriate target population.

Our simulations demonstrate that overlooking SAB—and more importantly, their differentiability by abruption and abnormal placentation status—raises concerns about left truncation bias for the effect of abruption on mortality on both the absolute and relative scales of measurement. It is essential to point out that in these simulated data, bias was greater and more variable among abnormal placentations because left truncation by SAB (C) was more common among abnormal placentations (*Z*, randomly generated in the first step of the simulation setup), driven by two mechanisms shown in Figure 2: (i) edge *β*_1_ whereby abnormal placentation causes an increased risk of SAB, and (ii) a separate path along edges *α* and *γ* whereby abnormal placentation starts a cascade of increased risk for SAB through an increased risk for abruption. In comparison, bias from abruption following normal placentation was negligible. Further studies and conceptualisations of this bias in real‐life data sources—either with better measures or through highly informed plasmode simulations (i.e., leveraging some real data within a simulation study), or both—are needed to confirm or refute these observations.

The process of human reproduction occurs on a relatively short gestational continuum [43]. However, studying outcomes related to obstetrical conditions presents a challenge since the origins of a given complication (e.g., abruption) may remain unknown. This ‘selection’ in pregnancy studies (cohort entry at ≥ 20 weeks) has been characterized as bias due to left truncation or, in other instances, as a ‘live birth’ bias [44, 45]. In this context, (unobservable) events that occur before 20 weeks are weeded out of the cohort, thereby introducing the notion of ‘depletion of susceptible’ and resulting in the ‘healthy survivor bias,’ a form of selection bias [45, 46, 47, 48].

The issue of left truncation has been the topic in other studies in perinatal epidemiology. In a simulation study, Howards et al. [19] showed that an analysis naïve to left truncation could bias the estimated odds ratio of exposure to trihalomethanes and the risk of SAB by 20% or more when the average gestational age at entry for the exposed versus the unexposed differed by 10 days or more. Through extensive simulations, Schisterman et al. [22] showed that (i) fixed or variable nondifferential left truncation results in a loss of precision; (ii) fixed or variable differential left truncation results in a bias either towards or away from the null as well as a loss of precision. The paradoxical inverse association between maternal smoking and preeclampsia was hypothesised to be the consequence of left truncation bias due to differential rates of early pregnancy loss among smokers. Through a simulation framework, Lisonkova and Joseph [21] showed that this hypothesis holds. This study was subsequently challenged by Kinlaw et al. [20], who showed that, under more plausible assumptions in the simulation models, left truncation does not appear to fully explain away the paradoxical (inverse) association between smoking and preeclampsia. Bruckner et al. [48] demonstrated another application of the left truncation bias to test among extremely preterm births whether the incidence of early neonatal death varies inversely with the incidence of stillbirth. They concluded that selection in utero may influence the survival characteristics of the live‐born cohort.

## 5 Conclusions

Defining the causal question regarding the abruption–mortality association should consider the target population, which may include all conceptions. Attempts to answer this causal effect by restricting the study to 20 weeks and beyond will likely introduce bias due to left truncation. This study underscores that the efforts to accurately define the target population to answer the causal question are paramount. Studies evaluating the total effect of abruption on mortality are biased if a ‘birth cohort’ design is adopted. Attempts to resolve (or at least minimise) the bias are best dealt with by considering a ‘conception cohort’ approach or through simulation‐based (or plasmode‐simulation‐based) quantitative bias analysis to explore the magnitude and direction of potential left truncation bias. We do not exclusively support a prescribed analytic approach to account for left truncation, but rather, the approach should be guided by the causal question. In a broader context, this study points to the inherent problems with abnormal placentation and the devastating consequences on maternal and offspring health along the life course.

## Author Contributions

C.V.A. conceived the project idea. A.C.K. developed and implemented the SAS programs and undertook the simulation analysis. A.C.K. and C.V.A. co‐wrote the paper. All authors reviewed the paper, provided comments for improvement, and approved the final submission.

## Ethics Statement

The authors have nothing to report.

## Conflicts of Interest

The authors declare no conflicts of interest.

## Supporting information


**Figure S1** Parameter combinations regarding the prevalence of abnormal placentation, risk of spontaneous abortion (*C*), and risk of perinatal mortality (*Y*), which includes stillbirth (at ≥ 20 weeks) or neonatal death (within the first 28 days) across simulation setups in the primary analysis (setups 1–10).
**Figure S2** Parameter combinations regarding the prevalence of abnormal placentation (*Z*), risk of spontaneous abortion (*C*), and risk of perinatal mortality (*Y*), which includes stillbirth (at ≥ 20 weeks) or neonatal death (within the first 28 days) across simulation setups in the primary analysis (setups 1–10) and sensitivity analysis (setups 11–40).
**Figure S3** Causal diagram representing associations in the simulated data between abnormal placentation (*Z*), placental abruption onset (*X*), spontaneous abortion (*C*), placental abruption diagnosis (*M*) and in utero death (stillbirth; *S*). Panel A represents the unobservable data in which spontaneous abortions are counted; panel B represents classically observable data in which investigators implicitly condition on spontaneous abortion by restricting a study to pregnancies lasting at least 20 weeks’ gestation. Conditioning on spontaneous abortion can induce collider bias and alter the ability to estimate the total effect of an exposure on an outcome (rather than estimating decomposed effects which require other nuanced assumptions) SAB, spontaneous abortion (10–19 weeks); SB, stillbirth (20–42 weeks).
**Figure S4**. Parameter combinations regarding the prevalence of abnormal placentation (*Z*), risk of spontaneous abortion (*C*) and risk of in utero death (stillbirth; *S*), which includes spontaneous abortion or stillbirth, across simulation setups that otherwise resemble the primary analysis (setups 41–50 resemble 1–10) and the sensitivity analyses (setups 51–80 resemble 11–30).
**Figure S5** Risks of perinatal mortality (*Y*), which includes stillbirth (at ≥ 20 weeks) or neonatal death (within the first 28 days) (panel A), risk differences (panel B) and risk ratios (panel C), stratified by abnormal placentation status across simulation setups in the primary analysis (setups 1–10) and sensitivity analysis (setups 11–40).
**Figure S6** Bias estimates: Bias in the risk of perinatal mortality (*Y*), which includes stillbirth(at ≥ 20 weeks) or neonatal death (within the first 28 days) (panel A), risk differences on the absolute scale (panel B), risk differences on the relative scale (panel C) and risk ratios (panel D) across simulation setups in the primary analysis (setups 1–10) and sensitivity analysis (setups 11–40).
**Figure S7** Risks of in utero death (stillbirth; *S*) (panel A), risk differences (panel B) and risk ratios (panel C), stratified by abnormal placentation status across simulation setups 41–80.
**Figure S8** Bias in risks of in utero death (stillbirth; *S*) (panel A), risk differences (panel B) and risk ratios (panel C) across simulation setups 41–80.

## Data Availability

The SAS program and design spreadsheet for the Monte Carlo simulations are available at https://github.com/alankinlaw/left‐truncation.
