# Supplementary material for: Placental Abruption and Perinatal Mortality: Abnormal Placentation and Spontaneous Abortion as Contributors to Left Truncation Bias
Source: Paediatr Perinat Epidemiol. 2025 Jun 5;40(2):133–43. doi: 10.1111/ppe.70010 (PMC12353283; doi:10.1111/ppe.70010)

**Supplemental Material**

**Placental Abruption and Perinatal Mortality:**

**Spontaneous Abortion as a Contributor to Left Truncation Bias**

Alan C. Kinlaw, Hillary L. Graham, Cande V. Ananth

**Supplemental figures**

| **Figures** | **Title** |
| --- | --- |
|  |  |
| **Figure S1** | Parameter combinations regarding the prevalence of abnormal placentation (*Z*), risk of spontaneous abortion (*C*), and risk of perinatal mortality (*Y*), which includes stillbirth, or neonatal death across simulation setups in the primary analysis (setups 1-10) |
|  |  |
| **Figure S2** | Parameter combinations regarding the prevalence of abnormal placentation (*Z*), risk of spontaneous abortion (*C*), and risk of perinatal mortality (*Y*), which includes stillbirth, or neonatal death across simulation setups in the primary analysis (setups 1-10) and sensitivity analysis (setups 11-40) |
|  |  |
| **Figure S3** | Causal diagram representing associations in the simulated data between abnormal placentation (*Z*), placental abruption onset (*X*), spontaneous abortion (*C*), placental abruption diagnosis (*M*), and *in utero* death (stillbirth; *S*)  ***Legend***: Panel A represents the unobservable data in which spontaneous abortions are counted; panel B represents classically observable data in which investigators implicitly condition on spontaneous abortion by restricting a study to pregnancies lasting at least 20 weeks’ gestation. Conditioning on spontaneous abortion can induce collider bias and alter the ability to estimate the total effect of an exposure on an outcome (rather than estimating decomposed effects which require other nuanced assumptions)  SAB, spontaneous abortion (10-19 weeks); SB, stillbirth (20-42 weeks) |
|  |  |
| **Figure S4** | Parameter combinations regarding the prevalence of abnormal placentation (*Z*), risk of spontaneous abortion (*C*), and risk of *in utero* death (stillbirth; *S*), across simulation setups that otherwise resemble the primary analysis (setups 41-50 resemble 1-10) and the sensitivity analyses (setups 51-80 resemble 11-30) |
|  |  |
| **Figure S5** | Risks of mortality (*Y*), which includes stillbirth or neonatal death (panel A), risk differences (panel B), and risk ratios (panel C), stratified by abnormal placentation status across simulation setups in the primary analysis (setups 1-10) and sensitivity analysis (setups 11-40) |
|  |  |
| **Figure S6** | Bias estimates: Bias in the risk of mortality (Y), which includes stillbirth or neonatal death (panel A), risk differences on the absolute scale (panel B), risk differences on the relative scale (panel C), and risk ratios (panel D) across simulation setups in the primary analysis (setups 1-10) and sensitivity analysis (setups 11-40) |
|  |  |
| **Figure S7** | Risks of *in utero* death (stillbirth; *S*) (panel A), risk differences (panel B), and risk ratios (panel C), stratified by abnormal placentation status across simulation setups 41-80 |
|  |  |
| **Figure S8** | Bias in risks of *in utero* death (stillbirth; *S*) (panel A), risk differences (panel B), and risk ratios (panel C) across simulation setups 41-80 |
|  |  |

**Figure S1**

**Parameter combinations regarding the prevalence of abnormal placentation (*Z*), risk of spontaneous abortion (C), and risk of perinatal mortality (Y), which includes stillbirth or neonatal death, across simulation setups in the primary analysis (setups 1-10)**


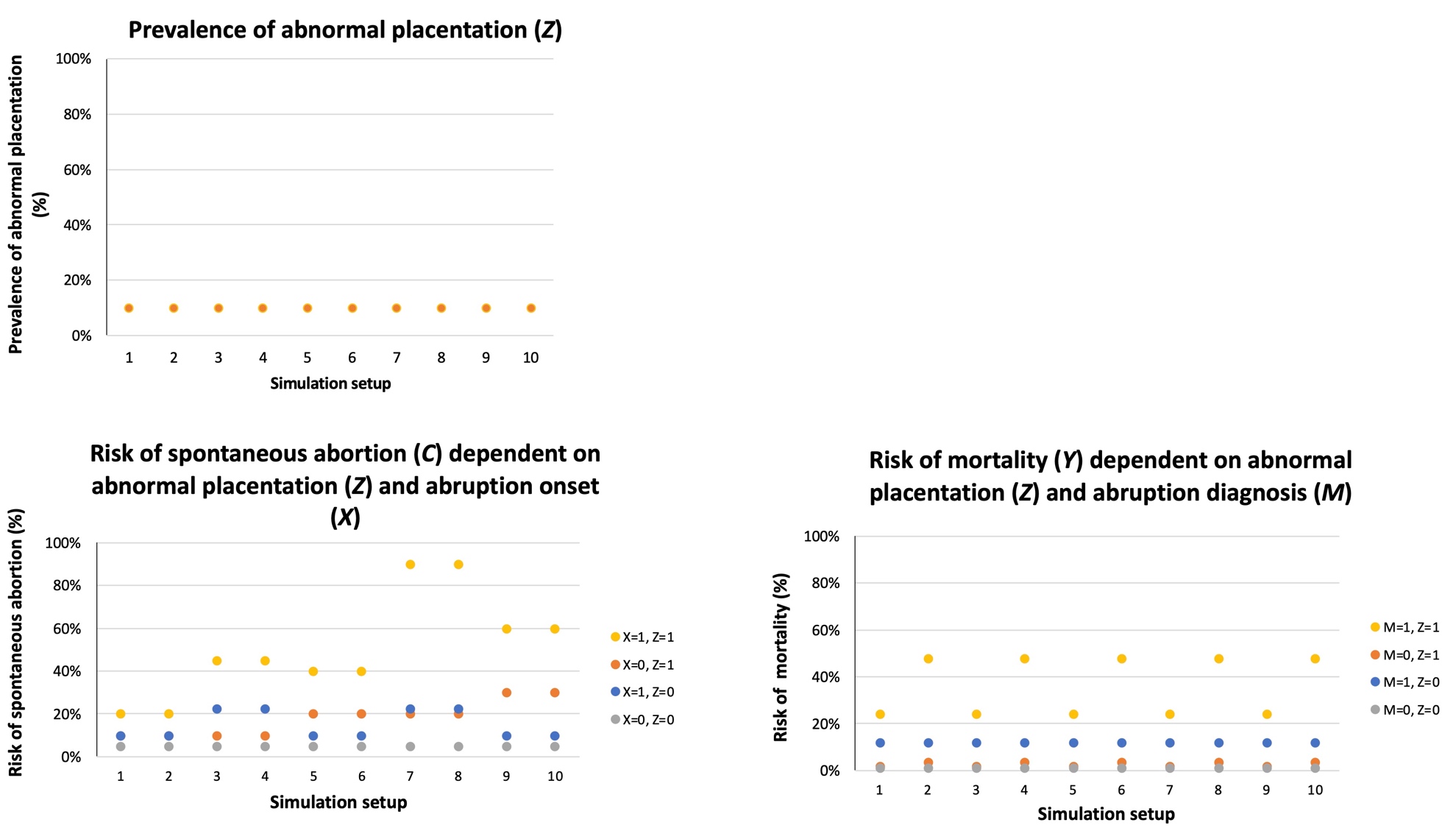


**Figure S2**

**Parameter combinations regarding the prevalence of abnormal placentation (*Z*), risk of spontaneous abortion (*C*), and risk of perinatal mortality (*Y*), which includes stillbirth or neonatal death, across simulation setups in the primary analysis (setups 1-10) and sensitivity analysis (setups 11-40)**


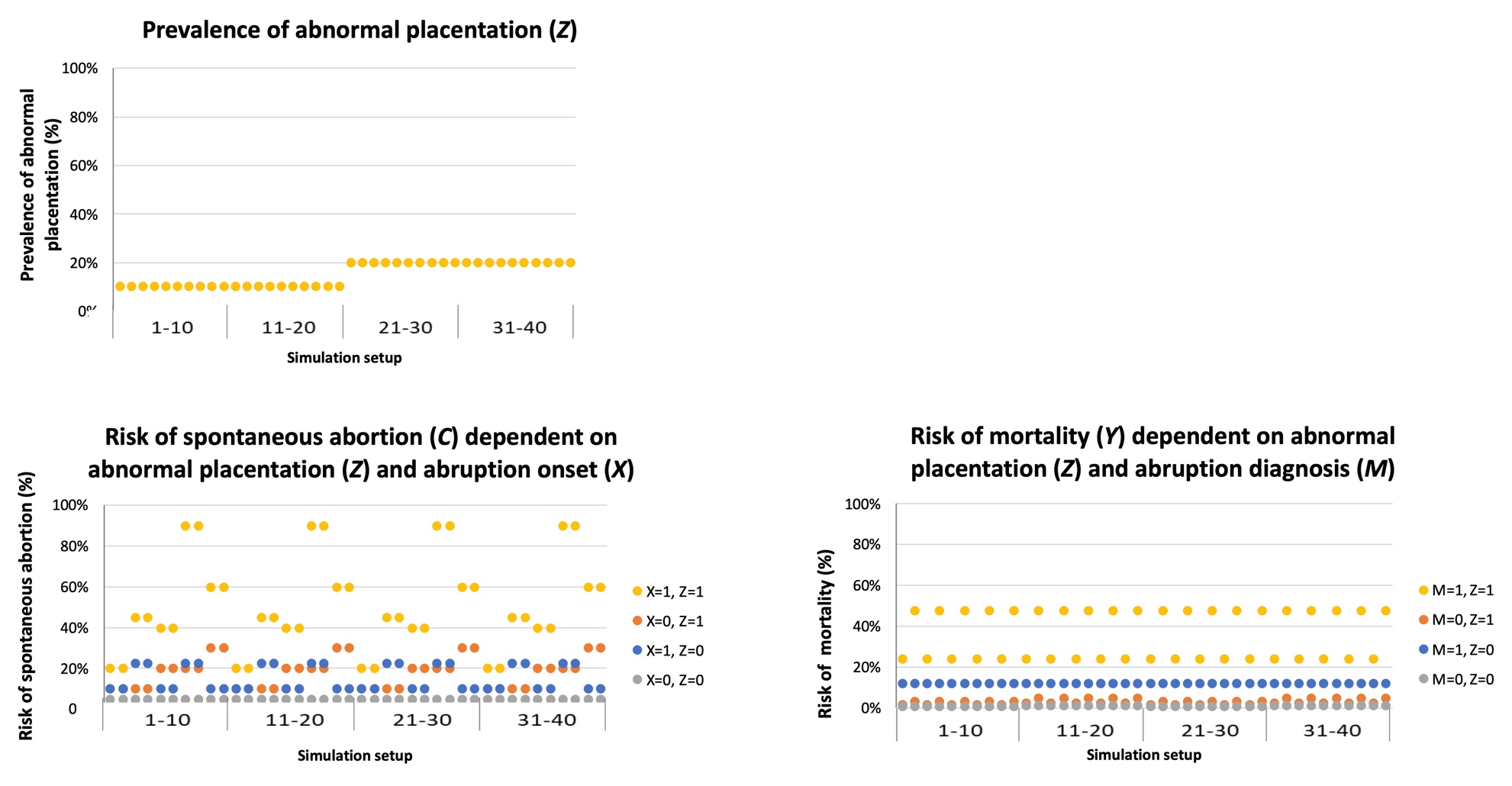


**Figure S3**

**Causal diagram representing associations in the simulated data between abnormal placentation (*Z*), placental abruption onset (*X*), spontaneous abortion (*C*), placental abruption diagnosis (*M*), and in utero death (stillbirth; *S*)**


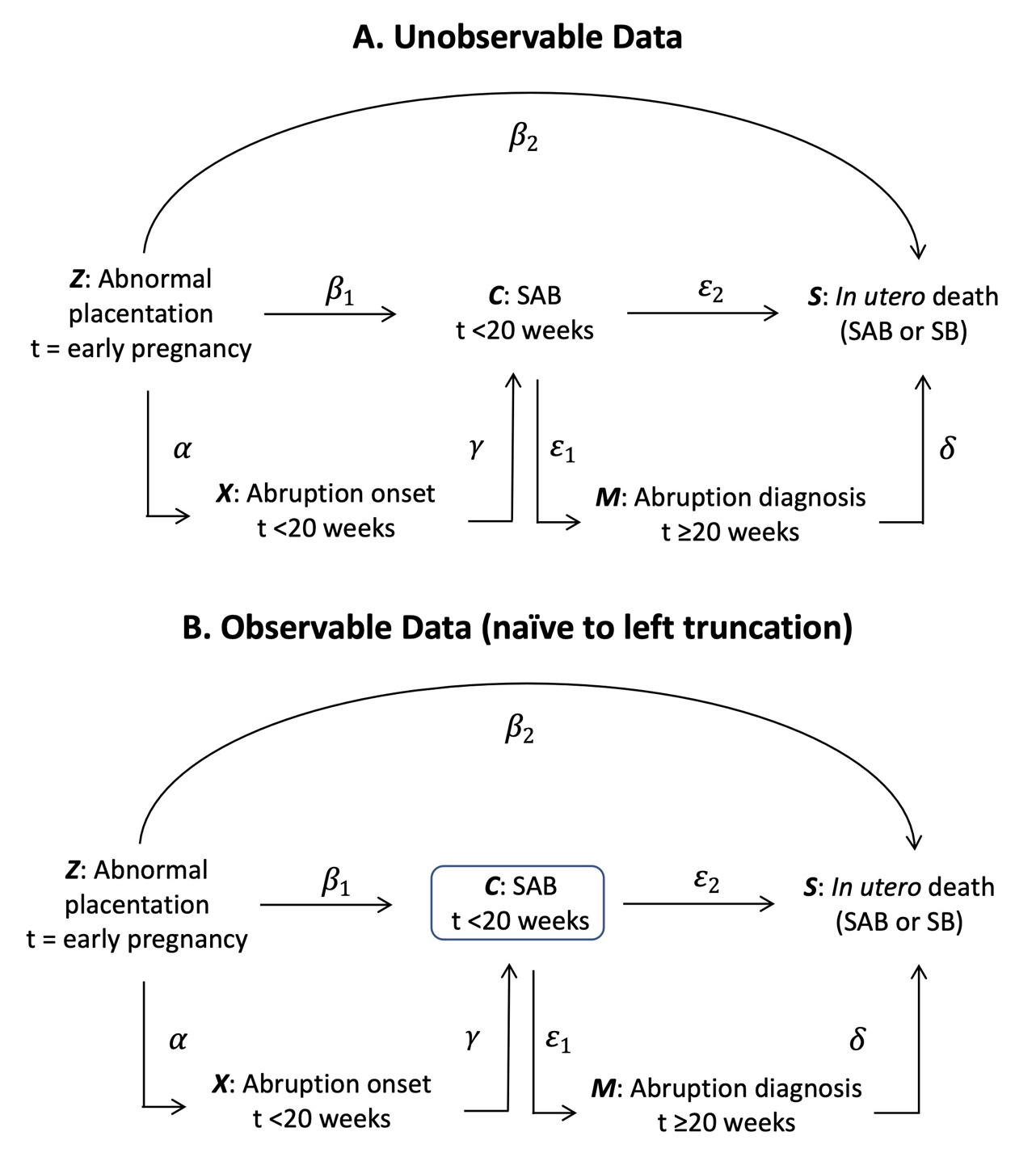


(stillbirth)

(stillbirth)

**Figure S4**

**Parameter combinations regarding the prevalence of abnormal placentation (*Z*), risk of spontaneous abortion (*C*), and risk of in utero death (*S*), which includes stillbirth, across simulation setups that otherwise resemble the primary analysis (setups 41-50 resemble 1-10) and the sensitivity analyses (setups 51-80 resemble 11-30)**


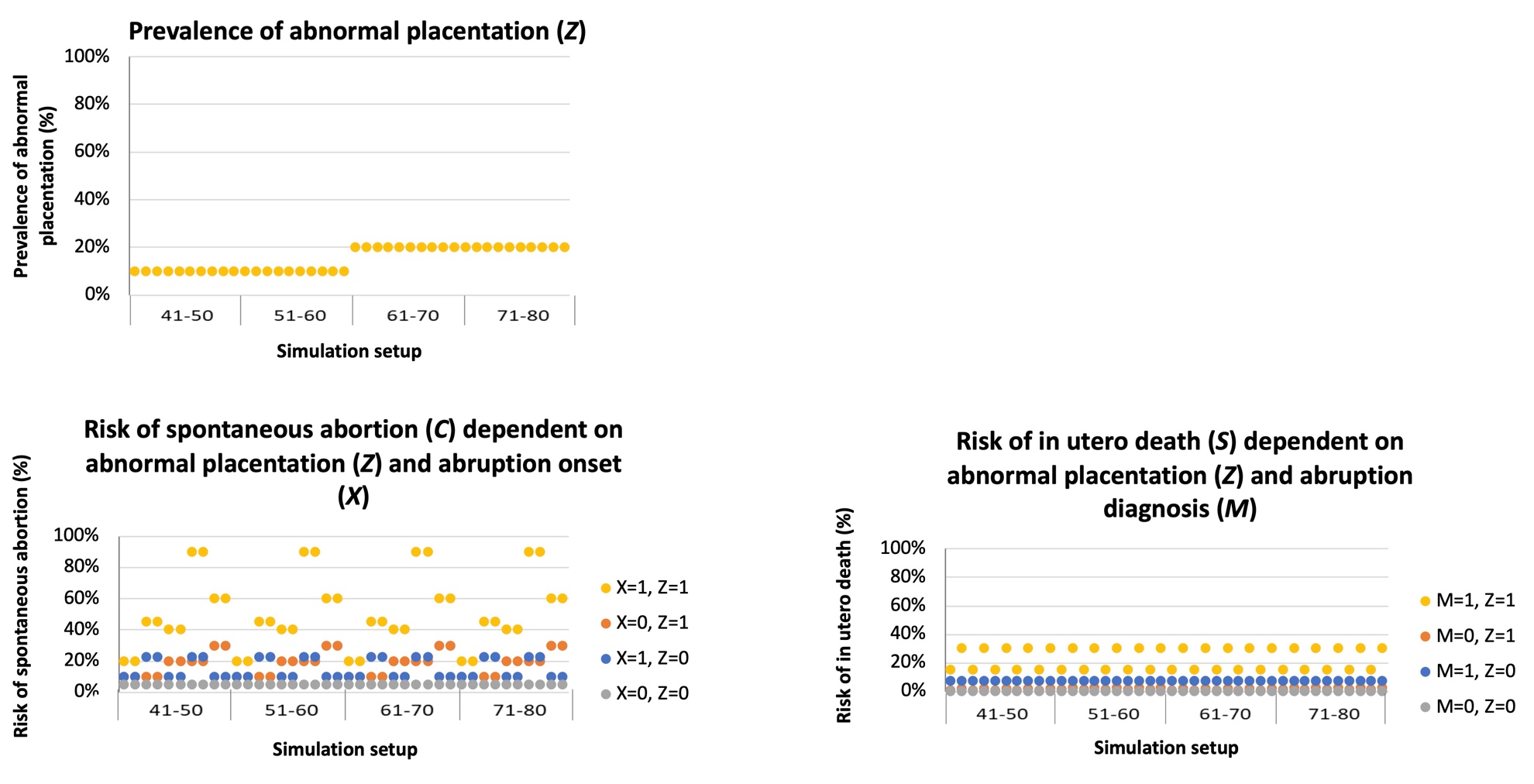


**Figure S5**

**Risks of mortality (*Y*), which includes stillbirth or neonatal death (panel A), risk differences (panel B), and risk ratios (panel C), stratified by abnormal placentation status across simulation setups in the primary analysis (setups 1-10) and sensitivity analysis (setups 11-40)**


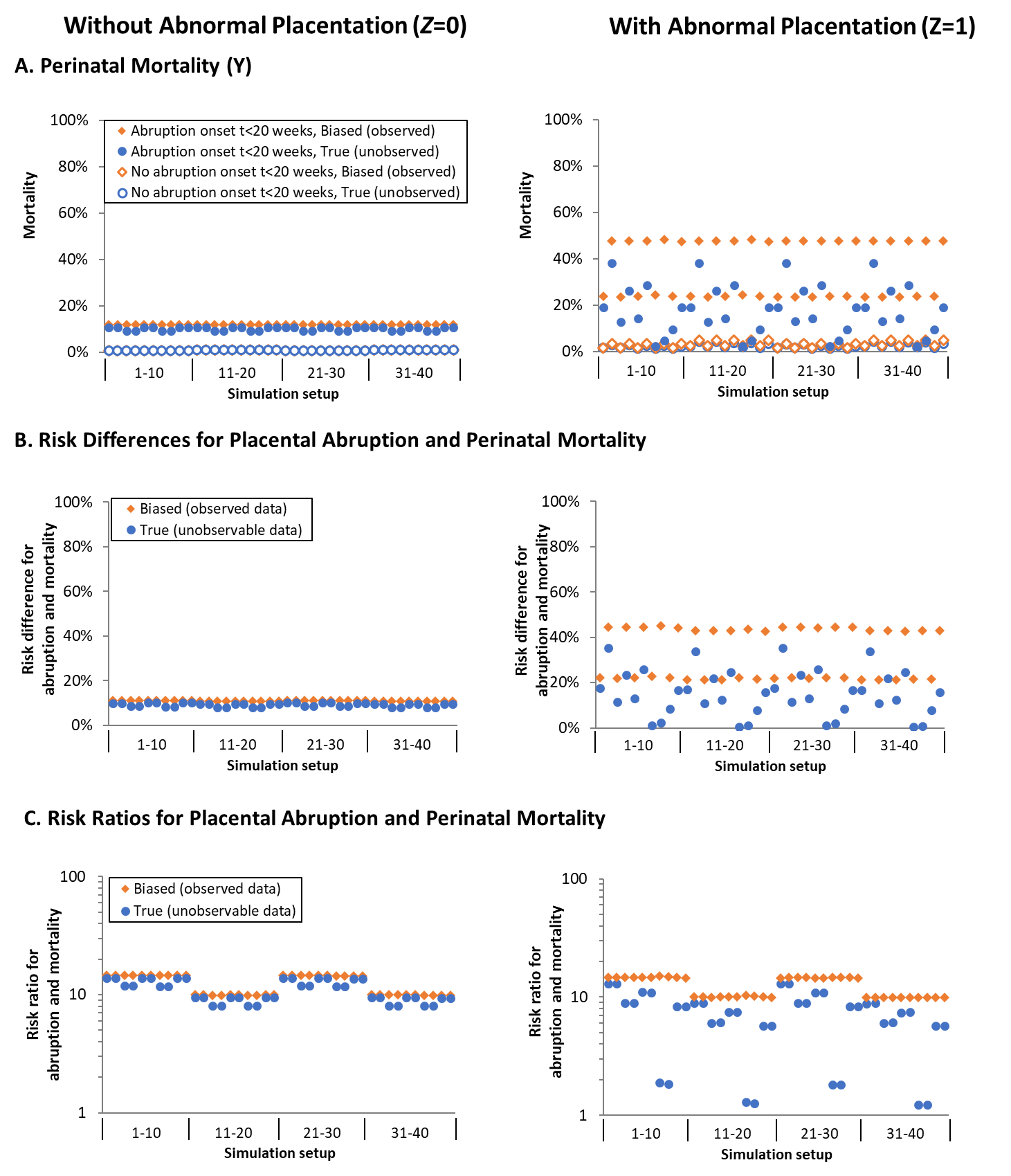


**Figure S6**

**Bias estimates: Bias in the risk of mortality (Y), which includes stillbirth or neonatal death (panel A), risk differences on the absolute scale (panel B), risk differences on the relative scale (panel C), and risk ratios (panel D) across simulation setups in the primary analysis (setups 1-10) and sensitivity analysis (setups 11-40)**


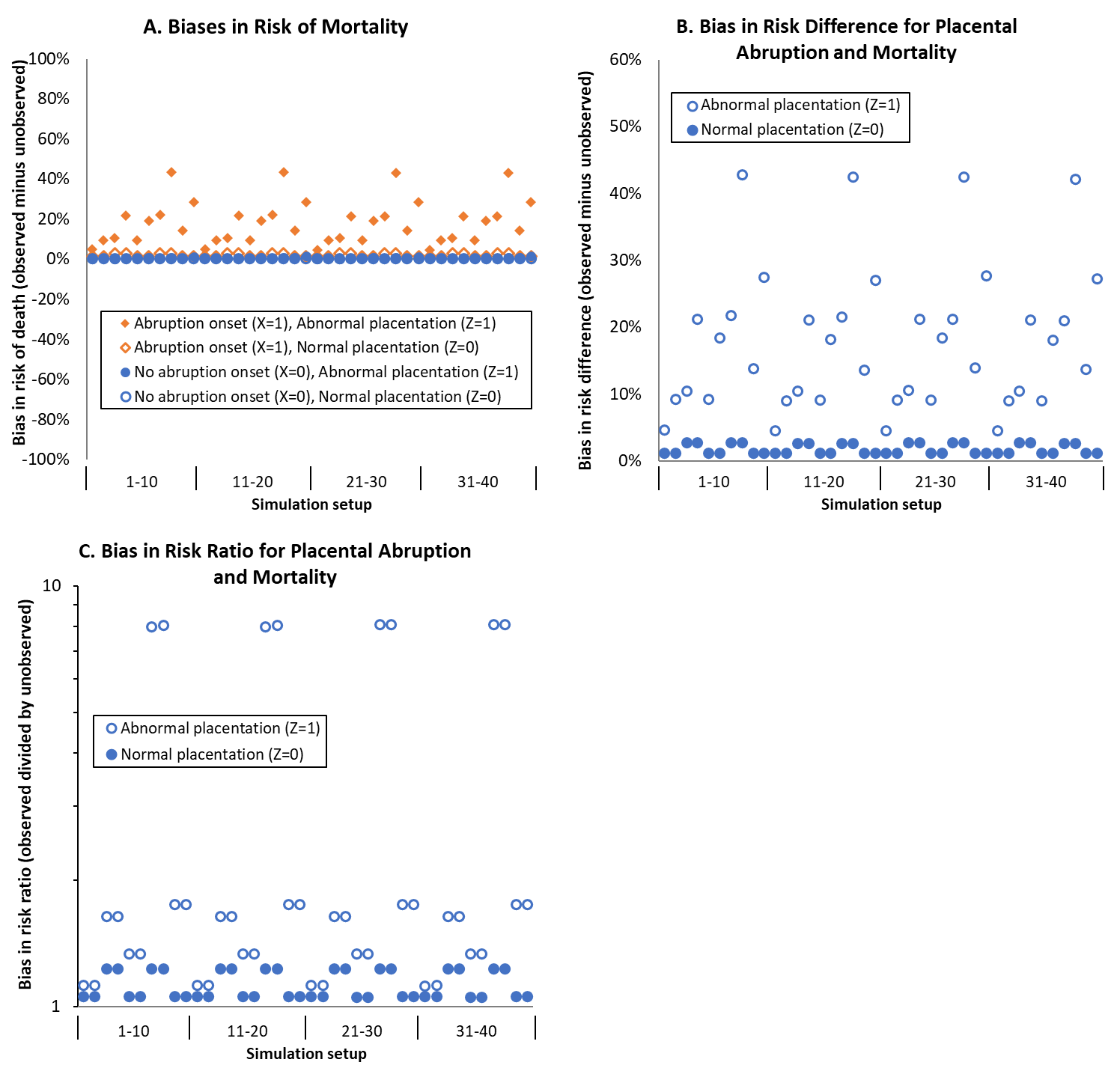


**Figure S7**

**Risks of *in utero* death (stillbirth; *S*) (panel A), risk differences (panel B), and risk ratios (panel C), stratified by abnormal placentation status across simulation setups 41-80**


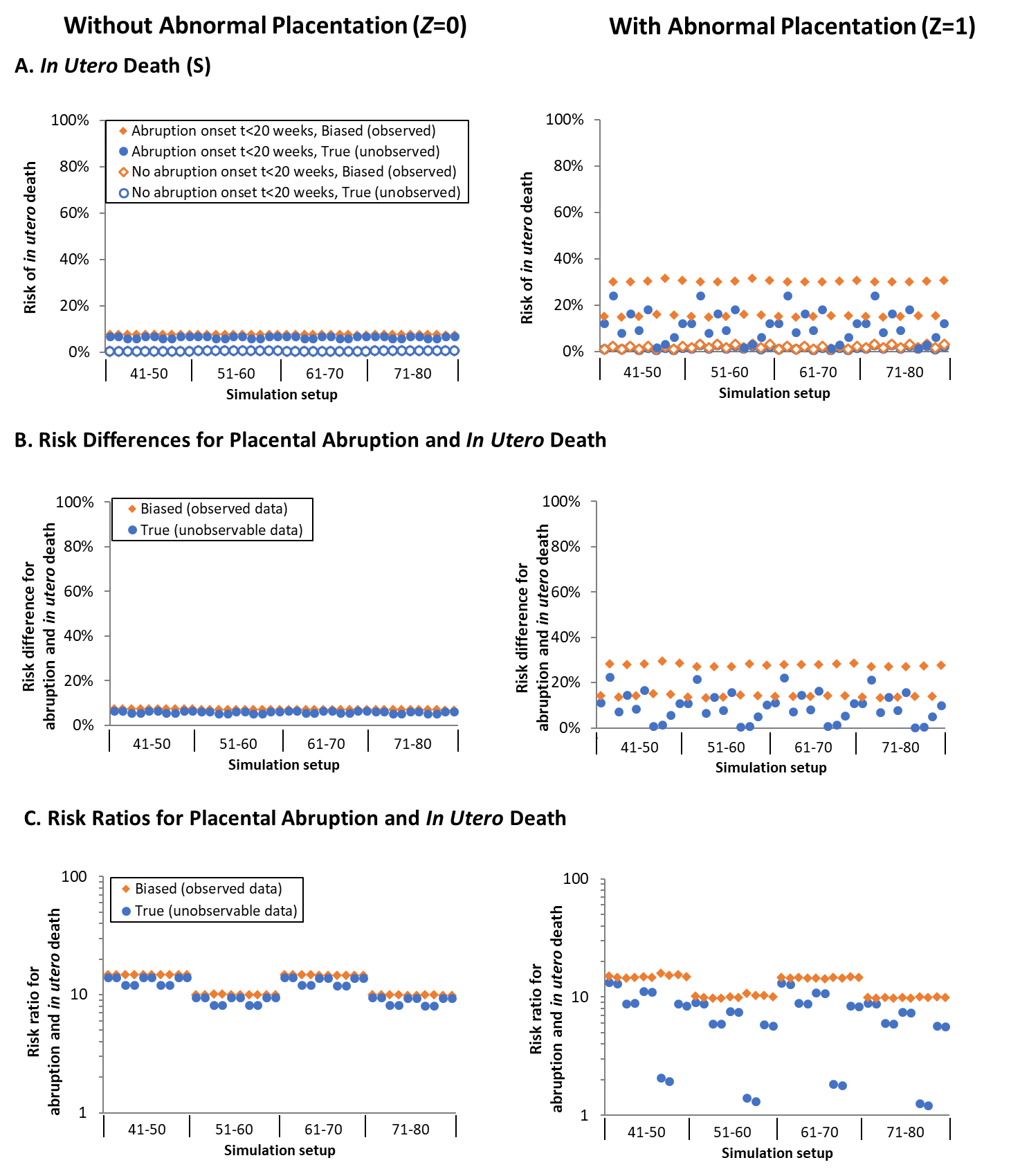


**Figure S8**

**Bias in risks of *in utero* death (stillbirth; *S*) (panel A), risk differences (panel B), and risk ratios (panel C) across simulation setups 41-80**


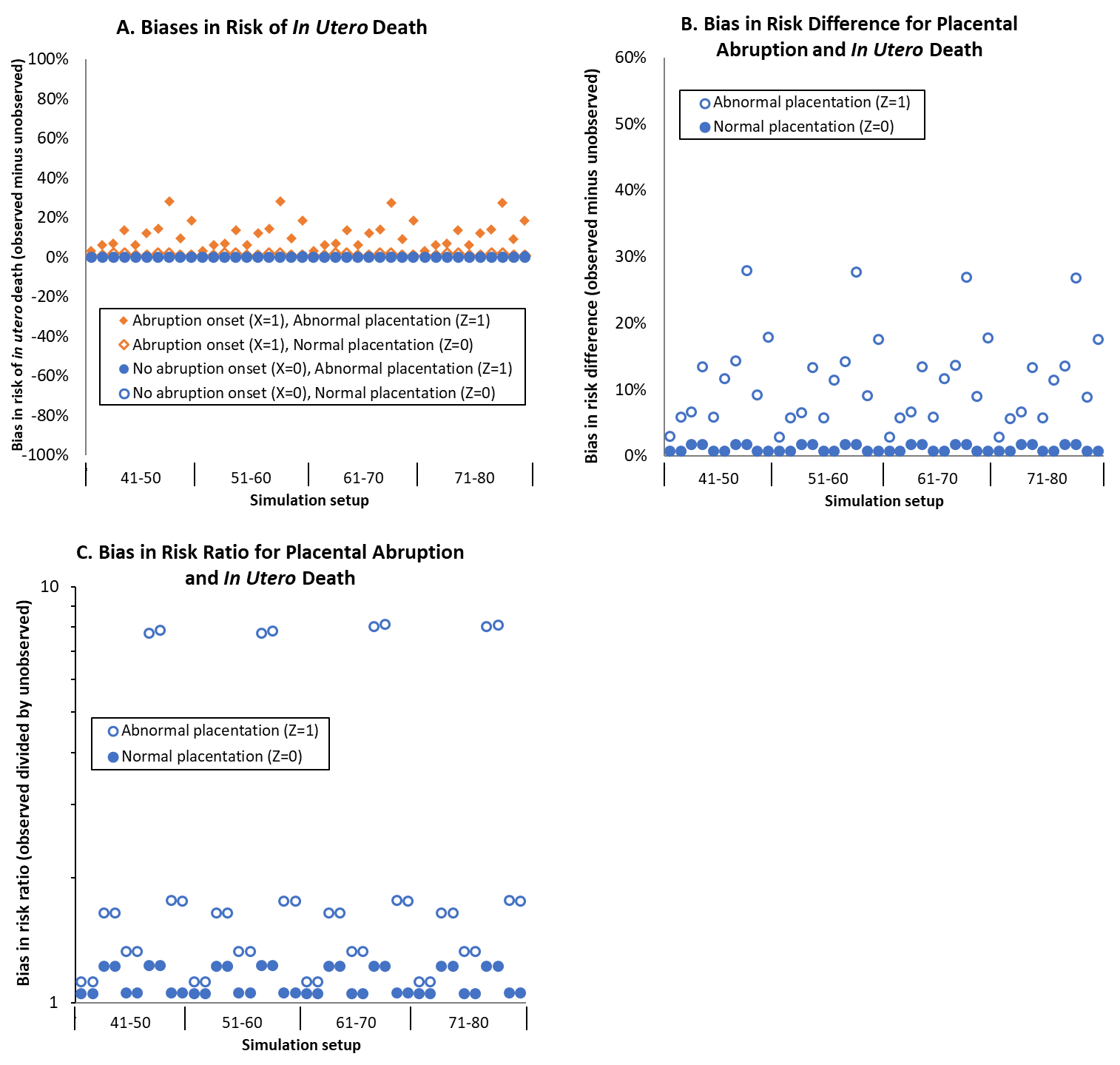

Supplement: Supplementary file 1 — Figure S1 Parameter combinations regarding the prevalence of abnormal placentation, risk of spontaneous abortion (C), and risk of perinatal mortality (Y), which includes stillbirth (at ≥ 20 weeks) or neonatal death (within the first 28 days) across simulation setups in the primary analysis (setups 1–10). Figure S2 Parameter combinations regarding the prevalence of abnormal placentation (Z), risk of spontaneous abortion (C), and risk of perinatal mortality (Y), which includes stillbirth (at ≥ 20 weeks) or neonatal death (within the first 28 days) across simulation setups in the primary analysis (setups 1–10) and sensitivity analysis (setups 11–40). Figure S3 Causal diagram representing associations in the simulated data between abnormal placentation (Z), placental abruption onset (X), spontaneous abortion (C), placental abruption diagnosis (M) and in utero death (stillbirth; S). Panel A represents the unobservable data in which spontaneous abortions are counted; panel B represents classically observable data in which investigators implicitly condition on spontaneous abortion by restricting a study to pregnancies lasting at least 20 weeks’ gestation. Conditioning on spontaneous abortion can induce collider bias and alter the ability to estimate the total effect of an exposure on an outcome (rather than estimating decomposed effects which require other nuanced assumptions) SAB, spontaneous abortion (10–19 weeks); SB, stillbirth (20–42 weeks). Figure S4. Parameter combinations regarding the prevalence of abnormal placentation (Z), risk of spontaneous abortion (C) and risk of in utero death (stillbirth; S), which includes spontaneous abortion or stillbirth, across simulation setups that otherwise resemble the primary analysis (setups 41–50 resemble 1–10) and the sensitivity analyses (setups 51–80 resemble 11–30). Figure S5 Risks of perinatal mortality (Y), which includes stillbirth (at ≥ 20 weeks) or neonatal death (within the first 28 days) (panel A), risk differences ( [file PPE-40-133-s001.docx]
